# Short-term transcriptional memory and association-forming ability of tomato plants in response to ultrasound and drought stress stimuli

**DOI:** 10.1080/15592324.2025.2556982

**Published:** 2025-09-12

**Authors:** Dóra Farkas, Anita Király, Viktor Ambrus, Bianka Tóth, Judit Dobránszki

**Affiliations:** a Centre for Agricultural Genomics and Biotechnology, Faculty of the Agricultural and Food Science and Environmental Management, University of Debrecen, Debrecen, Hungary; b Department of Biotechnology and Microbiology, Faculty of Science and Technology, University of Debrecen, Debrecen, Hungary

**Keywords:** Associative memory, conditioning, DNA methylation, mRNA-seq, *Solanum lycopersicum* L., sustained induction, training

## Abstract

Plant memory is an adaptive mechanism that plants can use to increase their fitness and cope with adverse environmental stresses. In this study, mRNA-sequencing (mRNA-seq), whole-genome bisulfite sequencing (WGBS) and real-time quantitative PCR (RT-qPCR) methods were applied for evaluating formation and maintenance of somatic transcriptional memory after treatment with ultrasound and drought stimuli in tomatoes. In addition, the effects of repeated stimuli, as well as the association-forming ability of plants were studied when they were trained previously with combined stimuli. Two days after exposure to the two stimuli applied alone or in combination, significantly altered gene transcription and DNA methylation were revealed. Using four selected target genes, we demonstrated that plants memorized stimuli for 5–10 d, in a gene- and stimulus-dependent way. The repeated application of the stimuli caused various alterations in gene transcription behavior, such as habituation, sustained induction or modified reinduction. Plants were able to use one conditioned stimulus as a predictor of the other, unconditioned one, after conditioning in the case of 3 out of 4 target genes, and used their transcriptional memory associatively. The exploitation of plant memory and associative learning may contribute to the development of new strategies to increase plant stress resilience.

## Introduction

The increasingly unpredictable environmental variability resulting from global climate change has a direct and growingly severe impact on the stability of agricultural production, plant stress tolerance and yield. Owing to their sedentary lifestyle, plants are unable to avoid adverse environmental conditions, such as drought or heat stress, and therefore, for their survival, they activate complex physiological and molecular adaptation mechanisms that help them adjust to environmental stressors.^1^^,^^2^^,^^3^

 Due to rising average temperature and increasing extremes of precipitation distribution, plants are forced to develop between successive abiotic stresses, which can have a significant negative impact on crop yield and quality.^4^ With the increasing incidence of extreme weather events, understanding plant stress responses and their molecular and epigenetic regulation is becoming increasingly urgent.

 There is accumulating theoretical and experimental evidence that the cognitive abilities of plants (perception, memory, and learning) cannot be completely excluded and in fact, their adaptive responses to some environmental stimuli may indicate a certain degree of learning.^5^ This learning-like phenomenon is supported, for example, by the fact that *Arabidopsis thaliana* L. can recognize the sounds produced by caterpillars and produces defensive toxins in response.^6^ *Mimosa pudica* L. gradually reduces its leaf-closing response to repeated touch, which can be interpreted as a form of simple habituation.^7^ Similar adaptive responses have been described in *Pisum sativum* L., which responds to the dynamics of nutrient distribution by altering its root architecture.^8^ In some cases, earlier milder stresses can ‘prepare’ plants for later, more severe stresses, a process called priming.^1^^,^^9^^,^^10^ Primed plants are able to respond faster and more effectively to recurring stress, and in some cases these responses can even be inherited for generations through epigenetic modifications (DNA methylation, histone modification).^1^^,^^4^,^11^^,^^12^ Zhang et al.^13^ investigated whether *Glechoma longitudo* L. plants can remember previous environmental stressors, such as UV-B radiation, and whether this memory affects the growth of progeny plants. The research demonstrated that clonal plants can remember environmental stress, epigenetically record the experienced stimulus (by decreasing DNA methylation), and transgenerationally transmit this memory to their progeny, influencing their defense strategies and growth.^13^ Recent research has indicated that transgenerational transmittance of acquired resistance is also possible in non-clonally propagated plants.^11^

According to our current knowledge, epigenetic modifications represent the deepest layer of plant memory.^12^ Although the blue-prints of plant memory are the epigenetic modifications,^12^ transcriptional memory is another layer of plant memory when, after a stimulus, the altered gene expression pattern is maintained compared to the naive state. This implies an altered transcriptional response after non-lethal stress, which is regulated by transient epigenetic changes (histone and DNA methylation modifications)^14^^,^^15^. A modified transcriptional response is a manifestation of plant memory. The physiological changes observed during priming include the activation of antioxidant enzymes, improved osmotic regulation, the activation of signaling pathways involved in stress responses, and increased production of secondary metabolites.^16^ The phenomena of priming and stress memory are primarily studied in model plant (*Arabidopsis thaliana* L.), while limited research results are currently available for agriculturally important crops (wheat (*Triticum aestivum* L.), rice (*Oryza sativa* L.), maize (*Zea mays* L.), rapeseed (*Brassica rapa* L.) or beans (*Phaseolus vulgaris* L.)).^17^^,^^18^ For example, in rapeseed, heat stress caused significant changes in the small RNA profile of pollen grains.^19^ In the heat-sensitive *P. vulgaris*, genetic association studies have identified several heat stress-responsive genes that affect various stress responses (heat shock protein (HSP) activation, flowering time regulation, signal transduction, and protein stabilization).^20^

The response mechanisms to environmental stressors differ by species and by stress type; therefore, species-specific studies are necessary. In connection with climate change, there is a growing demand for innovative and environmentally friendly technologies that can enhance the resilience of plants using memory processes induced by physical stimuli. Ultrasound stimulation may be an alternative solution as a new tool. Ultrasound/sound stimulation as an acoustic treatment is a partially explored research area; however, several studies have reported its beneficial physiological effects. Based on the studies conducted by López-Ribera and Vicient^21^ on the model plant *Arabidopsis thaliana*, sound treatment with white noise significantly increased the drought tolerance of plants and the expression of numerous key genes involved in several stress responses, including those responding to water deficit, oxidative stress and pathogens.^21^ Dobránszki et al.^22^ applied ultrasound treatment to single-node potato (*Solanum tuberosum* L.) explants in vitro, where the treatment was non-lethal and shoot growth was enhanced. According to their results, the acoustic stimulus functioned as a mild stress that induced adaptive growth responses.^22^

 Acoustic emissions from plants were first reported by Milburn and Johnson.^23^ They investigated that inside drought-stressed plants, water columns are under sufficient tension, which leads to cavitation, which causes a shock wave that can be detected as sound. This acoustic emission technique was used by Jackson and Grace,^24^ and they concluded that it can be used to determine the thresholds from which a plant's water-conducting system suffers damage, which environmental variables are harmful to the plant's water relations and which parts of the plant are most sensitive to cavitation, thus providing insight into drought tolerance. Ultrasonic acoustic emission was examined on the stems of mature field-grown trees of Scots pine (*Pinus sylvestris*) and pubescent oak (*Quercus pubescens*).^25^ In between emissions of the decibel (dB) range are often associated with cavitation, the prolonged emission of a lower dB range is also observed. High-decibel signals occurred at almost all drought intensities, but depending on the existing water deficit; under more moderate conditions when being well-watered than when they were under drought stress. Recently, Khait et al.^26^ discovered that tomato and tobacco plants exposed to various stresses (drought, cutting) emitted ultrasound in a range of 20–100 kHz. The emitted ultrasound was specific depending on to the type of the stress and could therefore be informative.

 When *Arabidopsis thaliana* plants were primed for plant immunity to *Sclerotinia sclerotiorum* by three repeated applications of sound stimuli (1 kHz sinusoidal waves at 100 dB). Hadj-Amor et al.^27^ revealed that there was a link between defense priming and the transcriptional memory of arabidopsis plants. This primed state was maintained by transcriptional stress memory through thousands of genes, probably regulated by transcription factor cascades.

An associative learning experiment was also conducted with *P. sativum* in a Y maze by Gagliano et al.^28^. In this study, an originally neutral cue (airflow) was paired with an instinctively preferred stimulus, i.e., light acted as an unconditioned stimulus, resulting in an unconditioned response, e.g., growing towards it. After training or conditioning, when only wind was applied, the plants grew in the direction previously associated with light, or in other cases, the plants avoided it, depending on whether the plants previously received the two stimuli from the same or opposite directions. The plants learned to associate wind (conditioned stimulus; CS) with light (unconditioned stimulus; UCS), and then, the CS (airflow) was enough to drive behavior (conditioned response; CR). Plants were not only able to use the once acquired information associatively, but they were able to recognize and form positive or negative associations both in space and time with the stimulus (light) in spite of prevalent light tropism in plants. According to Markel^29^, the results of the experiment could not be confirmed when repeated^29^. However, Gagliano et al.^30^ provided a simple explanation for this refutation: an experiment can be repeated only if the conditions remain the same. The different experimental conditions resulted in dissimilar results, therefore proven to be unsuitable for reproducing their experimental results^30^. In response to this explanation, Markel^31^ claimed that the differences in the experimental setups cannot cause a lack of replicability; however, considering the variations in materials and methods, Markel placed more emphasis on reproducibility. According to Cvrčková and Konrádová^32^, Markel attempted rather reproducibility than replicability of Gagliano’s experiment owing to differences in materials and methods. Up to date the only one study related to the existence of associative ability of plants at the transcriptional level came from Bhandawat et al.^33^. They used sound, such as UCS, to trigger a transcriptomic response to heat stress in *A. thaliana* and thereby up-regulation of heat-responsive genes were detected in conditioned plants in response to sound stimulus but without heat stress.

The aim of the present study was to explore the effects of ultrasonic (US) and drought (D) stimuli applied alone or in a combination on plant memory at different time points, i.e., 2, 5, 7, 10 and 12 d. We sought to answer the question of how long the gene transcription response mechanisms are sustainable. We also investigated whether repeated ultrasound or drought stimuli can strengthen the previously observed responsiveness, and how the timing of stimulus repetition affects responsiveness and memory stability. We also examined the mechanisms of action of combined treatments (using ultrasound and drought together), and whether plants can form an association between the two different stimuli, i.e., whether one stimulus (i.e., conditioned stimulus, CS) applied alone can elicit a response related to the other (i.e., unconditioned stimulus, UCS) if they were previously applied together^34^. We evaluated the stability of memory traces and the possibility of associative learning in terms of temporal persistence. Accordingly, we have examined the following main hypotheses: (1) 2 d after exposure to the two stimuli (D and US) applied alone or in combination, plants significantly altered gene transcription and DNA methylation, and a correlation between these changes can be shown; (2) treatment of plants with US or D stimuli applied separately or in combination was memorized by the plant for 5 or 10 d, thereby altering the expression intensity of selected target genes; (3) the repeated application of the two stimuli reinforces the plant’s transcriptional memory; (4) plants are able to connect different stimuli and use one conditioned stimulus as a predictor of the other, unconditioned stimulus, after training.

## Materials and methods

### Plant material and growing conditions

In the experiments, tomato (*Solanum lycopersicum* L. cv. Micro Tom) plants were used. Seeds of the tomato cv. Micro Tom were originated from Hungarian University of Agriculture and Life Sciences, Institute of Genetics and Biotechnology, and have been continuously propagated in our institute to obtain the starting materials necessary for the experiments.

 The Jiffy-7® discs were soaked with a solution containing MS macro and microelements^35^ and 0.15% Previcur Energy fungicide until they became peat cylinders ready for sowing. Tomato seeds were individually sown in Jiffy-7® peat cylinders (mentioned later as Jiffy), which were individually placed in a Veg-box and placed in a climate chamber (24 °C, photoperiod of 16/8 h day/night with a photosynthetic photon flux density (PPFD) of 89 µmol m^−2^ s^−1^, provided by a 1:1 ratio of warm white and daylight fluorescent lamps; the relative humidity was RH: 65%). The germinated seedlings were cultivated there for three weeks before the treatments. After each treatment, cultivation was continued under the same light, temperature and RH conditions; treated plants were placed randomly in the culture room. Jiffys were irrigated twice a week with 15 ml of tap water.

### Training and sampling procedures

**Figure 1.**
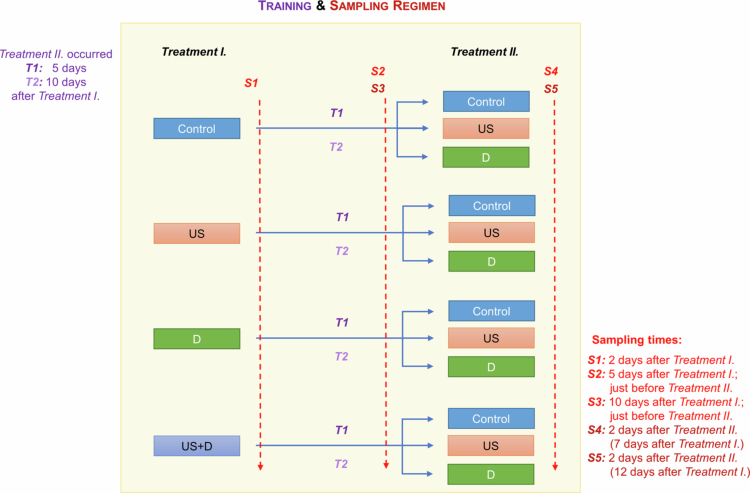
Schematic illustration of plant training (T) and sampling (S) regimen (abbreviations: US – ultrasound treatment; D – drought treatment; US + D – ultrasound and drought treatment).

Two stimuli were applied in the experiments, ultrasound and drought stress, separately and in combination. They were applied at the onset of the experiments (initial stimuli; Treatment I), and then again 5 and 10 d after the initial stimuli in the first and second series of experiments (Treatment II), respectively (Figure 1). Drought stress (D) was induced by irrigating each plant with 10 ml of a solution that contained 20% PEG (polyethylene glycol 6000). Ultrasonication (US) was performed with an ultrasonicator at 30 kHz and 120 W ultrasound treatment for 5 min when the average distance between the plants and the ultrasonic head was 6 cm, and the medium of transmission was air. In the combined treatment (US + D), the ultrasonicated plants were irrigated with the aforementioned PEG solution immediately after ultrasonication. A total of 168 plants were included in the experiments. Each initial treatment (D, US, US + D) and the control group consisted of 42 plants at the beginning (Treatment I), and 3 different groups were generated from each one and used for Treatment II. (control, US, and D).

 Changes in gene transcription and DNA methylation in response to US, D, and US + D treatments 48 h (2 d) after the treatments were investigated. The effect of repeated application (Treatment II, on day 5 or 10 after Treatment I) of the two stimuli (US and D) on gene expression was also studied 2 d after their applications (on the 7^th^ or 12^th^ day). After training with the combined treatment (US + D, Treatment I), in the associative part of the experiment, in Treatment II, ultrasound was used as a CS while drought stress as an UCS for 2 selected target genes, while in the case of the other 2 target genes, drought was a CS while ultrasound was an UCS. Their effects on changes in the expression intensity of selected target genes were studied (see below). Training was performed in two steps of conditioning: Treatment I at the beginning of the experiments (initial stimuli) and Treatment II on the 5^th^ or 10^th^ day of the experiments (repeated stimuli) in two independent experimental series. The training procedures and sampling regimens are summarized in Figure 1. The absolute control plants were not treated and were indicated with C at S1, S2, S3, while with ‘C and C’ at S4 and S5 sampling times. Besides, plants that only received treatments at Treatment I (i.e. non-recurrent stimuli), but not at Treatment II, were considered as controls of plants that received recurrent stimuli (Treatment II), as well. They were necessary to control for the possible contribution of the seedlings’ ongoing development, and they were indicated as ‘US and C’, ‘D and C’, and ‘US + D and C’.

There were five sampling times; at each sampling time, 3 different biological replications were collected randomly from each treatment or treatment combination. Sampling was carried out 2 d after the initial stimuli (Treatment I) (S1); 5 d (S2) or 10 d (S3) after the Treatment I, just before the repeated stimuli (Treatment II), and finally 2 d after the Treatment II in both sets of experiments (S4 and S5) (Figure 1).

### RNA and DNA isolation for sequencing

*Genomic DNA isolation:* DNA isolation from tomato leaves was performed in two biological replicates from each treatment and control groups from the first sampling time (S1, 2 d) according to the Macherey-Nagel Nucleospin® Plant II kit protocol. The concentration and purity of the extracted DNA were measured on NanoPhotometer® N50 (Implen, Munchen, Germany).

*Total RNA isolation:* Total RNA was extracted from tomato leaves in three biological replicates (S1, 2 d) using Quick-RNA™ Plant Miniprep Kit (Zymo Research, Irvine, CA, USA) according to the manufacturer’s protocol. RNA quantity and quality were assessed via microcapillary electrophoresis using a NanoPhotometer® N50 (Implen, Munchen, Germany), and by fragment analysis with an Agilent Bioanalyzer 2100 system using RNA 6000 Nano Kit (Agilent, Santa Clara, CA, USA).

### RNA sequencing (RNA-seq) and bioinformatics

mRNA library preparation, encompassing poly-A enrichment and cDNA library construction, and sequencing were conducted by Novogene. High-quality RNA samples were subjected to quality control using NanoDrop spectrophotometer for concentration and purity, agarose gel electrophoresis for integrity, and an Agilent 2100 BioAnalyzer for RNA Integrity Number (RIN) assessment. Following library preparation, paired-end 150 bp sequencing was performed on the Illumina NovaSeq X Plus Series platform (PE150).

 Raw sequencing reads were trimmed to remove adapters using fastp v0.26.0^36^ and quality-checked with FastQC v0.12.1^37^. Read alignment was performed using Hisat2 v2.1.1^38^ against the tomato reference genome (*S. lycopersicum* SL4.0, Sol Genomics Network). The aligned reads were quantified with FeatureCounts package v2.1.1^39^ using the ITAG4.0 annotation (Sol Genomics Network). Data normalization and clustering were conducted prior to extracting differentially expressed genes (DEGs) using DeSeq2 v1.44.0^40^. The threshold for DEGs was set at a log2-fold change > 0.58 and an adjusted *P*-value < 0.05.

### Whole-genome bisulfite sequencing (WGBS) and bioinformatics

DNA methylation library preparation, including DNA fragmentation and bisulfite treatment, and sequencing was carried out by Novogene. Genomic DNA was extracted, fragmented, and treated with sodium bisulfite to convert unmethylated cytosines to uracil, preserving methylated cytosines. Libraries were constructed following adapter ligation and amplification, with quality control performed to ensure sufficient yield and fragment size distribution. Sequencing was conducted using a paired-end 150 bp strategy on the Illumina NovaSeq X Plus Series platform (PE150).

 Raw sequencing reads were quality-checked using FastQC v0.12.1^37^ and filtered and trimmed with fastp v0.26.0^36^ software. Bismark v0.24.2^41^ was used to align the trimmed reads to the tomato reference genome (*S. lycopersicum* SL4.0, Sol Genomics Network). After deduplication of multi-aligned reads, methylation extraction was performed for CpG, CHH, and CHG contexts. Differentially methylated regions (DMRs) and differentially methylated genes (DMGs) were identified using MethylKit v1.30.0^42^, with a cytosine read coverage threshold of 10X and a significance threshold of *P* < 0.05. DMRs were defined using a 200-bp sliding window, with a methylation percentage difference greater than 10%. DMGs were identified as genes with DMRs located within the gene body and 3 kb upstream promoter regions. Annotation of DMRs and DMGs was performed using the Genomation package v1.36.0.^43^

Gene Ontology (GO) annotations for both DEGs and DMGs were retrieved from the Sol Genomics Network (https://solgenomics.net/) website. Functional enrichment analysis of DEGs and DMGs was conducted using the ClusterProfiler package v4.12.6^44^ to identify significantly enriched GO terms with a significance threshold of *P* < 0.05. The ggplot2 package v 3.5.2^45^ of the R programming language v4.4.1^46^ was used for visualization.

### RT-qPCR analysis

#### Total RNA isolation for RT-qPCR analysis

Total RNA was purified from three biological replicates (all of the leaves of the sampled plants) from each control and treated samples using Quick-RNA™ Plant Miniprep Kit (Zymo Research, Irvine, CA, USA) according to the manufacturer’s protocol. Plants from which samples were taken previously were excluded from further sampling. Two quality control methods were applied after purification. For the preliminary quantification, microcapillary electrophoresis with an Implen n50 nanophotometer (Implen, Munich, Germany) was used. To assess RNA degradation and potential contamination, an Agilent Bioanalyzer 2100 system with an RNA 6000 Nano Kit (Agilent, Santa Clara, CA, USA) was used.

#### cDNA synthesis

The cDNA was synthesized from 200 ng of RNA with 2.5 µM of Random nonamer using a Reverse Transcriptase Core kit (Eurogentec, Searing, Belgium) following the manufacturer’s instructions. The reaction consisted of an initial step of 25 °C for 10 min, a reverse transcriptase step of 48 °C for 30 min and inactivation of the RT enzyme at 95 °C for 5 min. All samples were stored at −20 °C until further analysis.

#### Primer design

Four reference/housekeeping genes were selected based on previous studies, they included exocyst complex component SEC3A (SEC), elongation factor 1-alpha (ELO), glyceraldehyde 3-phosphate dehydrogenase (GAPDH), and ubiquitin-ribosomal protein eS31 fusion protein-like (UBI)^47^^,^^48^. The comparative ΔCt method,^49^ geNorm,^50^ NormFinder,^51^ BestKeeper^52^ statistical methods were used along with RefFinder comprehensive tool^53^ to compare the stability of expression intensity among candidate housekeeping/reference/normalizing genes based on the cycle quantification (Cq) value (Supplementary Table 1).

 Candidate target genes were selected based on the most negative and positive logarithmic fold change values in RNA-seq gene expression data.

The sequences of the target and housekeeping genes were initially obtained from the National Center for Biotechnology Information (NCBI). All primers were designed using the software SnapGene (v8.0) and were also checked with PCR Primer Stats (www.bioinformatics.org) to evaluate potential primers. The primer design conditions were as follows: melting temperature (T_m_) of 56−62 °C, primer length of 18−25 base pairs (bp), GC content between 40% and 60%, and PCR product length of 150−250 bp. Each potential amplicon was tested for homology with other genes using NCBI BLASTn (Basic Local Alignment Sequence Tool for nucleotides). All primers were synthesized by Integrated DNA Technologies (Leuven, Belgium).

#### Validation of DEGS by RT-qPCR

For the validation, three biological replicates from each control and treated (S1, 2 d) samples were used in three technical replicates. Total RNA (200 ng) was reverse transcribed to cDNA using the Reverse Transcriptase Core kit (Eurogentec, Searing, Belgium). RT-qPCR was performed with the Takyon™ No Rox® SYBR MasterMix dTTP Blue kit (Eurogentec, Searing, Belgium) following the manufacturer’s protocol on AriaMx Real-Time PCR System (Agilent Technologies Inc., USA). Pearson correlation coefficient was calculated and illustrated using GraphPad Prism version 10.0.0 for Windows (GraphPad Software, Boston, Massachusetts, USA; www.graphpad.com).

#### RT-qPCR analysis

RT-qPCR were performed using the Takyon™ No Rox® SYBR MasterMix dTTP Blue kit (Eurogentec, Searing, Belgium) following the manufacturer’s protocol. The reaction mix included 2.5 µl of cDNA, 2 µl of each oligonucleotide, 10 µl of Takyon™ MasterMix and 3.5 µl of water to a final reaction volume of 20 µl. Each primer (reverse and forward) was used at a concentration of 0.1 µM. The amplification protocol consisted of a Takyon™ activation at 95 °C for 3 min and 40 cycles of 95 °C for 10 sec (denaturation), a primer-specific annealing temperature of 52.5 °C for 20 sec, and an extension of 72 °C for 30 sec. Three biological replicates were run in triplicates (three technical replicates) from each control and treated samples.

### Statistical analysis

For the RT-qPCR analysis, the mean of three technical replicates were used, then relative gene expression was subsequently calculated with the 2^(^^−ΔΔCq)^ method.^54^ The gene expression logarithmic fold change (LFC) was calculated along with standard deviations using Excel in (Microsoft, Redmond, WA, USA). Statistical analysis of the RT-qPCR results was performed with IBM SPSS Statistics 25 software (SPSS®, version 25.0). Two-way ANOVA followed by Tukey’s B-test was used for multiple comparisons. A value of *P* < 0.05 was considered statistically significant. LFC values were used to present the results using GraphPad Prism version 10.0.0 for Windows (GraphPad Software, Boston, Massachusetts, USA; www.graphpad.com).

## Results

### Transcriptional and DNA methylation changes in response to drought and ultrasound 2 d after their application

**Figure 2.**
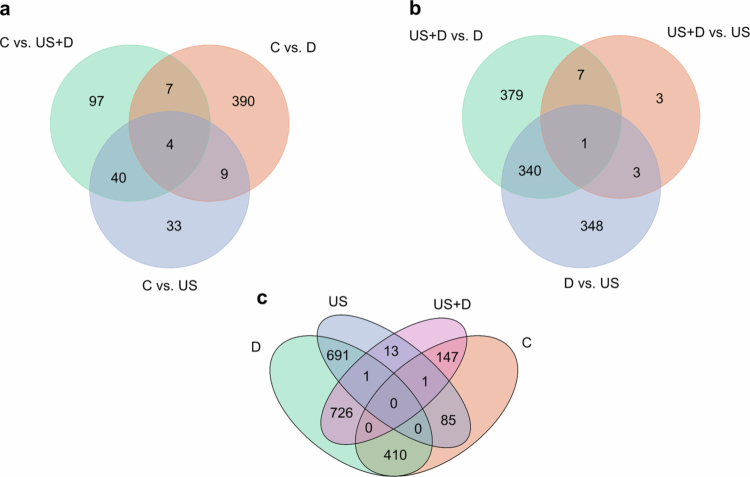
Venn diagram of DEGs (differentially expressed genes) (a) relative to the untreated control, (b) in pairwise comparisons between US + D, US, and D treatments, as well as (c) intersection of comparisons (abbreviations: US – ultrasound treatment; D – drought treatment; Us + D – ultrasound and drought treatment; C – control).

Global changes in mRNA expression profile of 34,075 genes revealed significantly up- or down-regulation of 86, 410, and 148 genes (differentially expressed genes, DEGs) compared to the control treatment when US, D, and combined (US + D) stimuli were applied, respectively (Figure 2a; Supplementary Figure 1; Supplementary Table 2). When the effects of the stress stimuli were compared pairwise with the control, 7, 9, and 40 DEGs were identical in response to US + D and D, D and US, and US and US + D, respectively. There were 4 DEGs that responded to all the treatments with significantly different expression (Figure 2a; Supplementary Table 2).

 Functional enrichment analysis for gene ontology (GO) categories, such as biological processes (BPs), cellular components (CCs), and molecular functions (MFs), was carried out using DEGs with a log2-fold change > 0.58 and *P*-value < 0.05. Most BPs were detected when D was compared to the control, DEGs related to 7 BPs, i.e., sucrose metabolic process, response to oxidative stress, interstrand cross-link repair, cellulose microfibril organization, cell growth, biosynthetic process and DNA replication. In case of US treatment, no BPs could be annotated while, regarding the combined treatment (US + D), the BP of response to wounding was identified. Cellular components were annotated only in the case of D treatment, they were the following 4 CCs: monolayer-surrounded lipid storage body, obsolete anchored component of membrane, Fanconi anaemia nuclear complex and obsolete integral component of peroxisomal membrane (Supplementary Table 3).

When the treatments were compared to each other pairwise, a total of 1,433 genes had altered their expression, 727 of which were altered in both comparisons between US + D vs. D and D vs. US, 692 between D vs. US and US + D vs. US, and 14 between US + D vs. US and US + D vs. D. 340 DEGs were identical in response to US + D vs. D and D vs. US, 3 in D vs. US and US + D vs. US, and 7 in US + D vs. US and US + D vs. D. Only one DEG was found that changed in each comparison pair (Figure 2b; Supplementary Table 2). Figure 2c summarizes the identical genes at the intersection of the comparisons.

Only 1−1 biological processes, carbohydrate metabolic process in US vs. D comparison and proteolysis in US vs. US + D comparison could be annotated (Supplementary Table 3). In US vs. D comparison, 3 CCs, i.e., the monolayer-surrounded lipid storage body, the Fanconi anaemia nuclear complex and the obsolete anchored component of plasma membrane, were related while, in addition to the Fanconi anaemia nuclear complex other 2 CCs, i.e., the 1,3-beta-D-glucan synthase complex, and the obsolete anchored component of the membrane, were revealed in the US + D vs. D comparison. When US was compared to US + D, only the intracellular anatomical structure was annotated (Supplementary Table 3). Molecular functions could be identified only in 2 comparisons. In US vs. D comparison, 2 MFs (hydrolase activity, hydrolyzing O-glycosyl compounds and transmembrane transporter activity) while in US vs. US + D comparison 4 MFs (aminopeptidase activity, malate dehydrogenase (decarboxylating) (NAD^+^) activity, DNA-binding transcription factor activity and sequence-specific DNA binding) were annotated (Supplementary Table 3).

**Figure 3.**
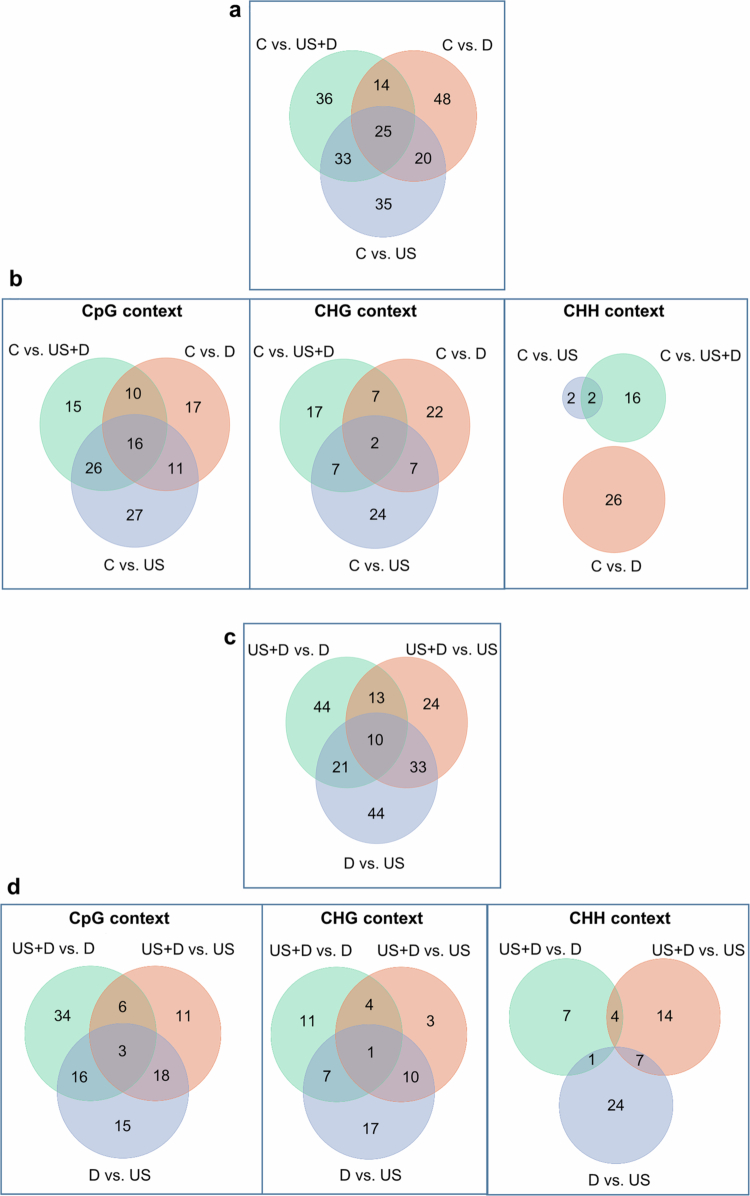
Venn diagram of DMGs (differentially methylated genes) in all contexts (a), as well as for 3 contexts (CpG, CHG and CHH) (b) relative to untreated control, and in all contexts (c), As well as for 3 contexts (CpG, CHG and CHH) (d) in comparisons between US + D, US and D treatments (abbreviations: US – ultrasound treatment; D – drought treatment; US + D – ultrasound and drought treatment; C – control).

Analysis of global DNA methylation at cytosine sites including both promoter and coding regions of 34,075 genes, revealed differentially methylated genes (DMGs); both hypo- and hypermethylation were detected (Supplementary Tables 4−6). A total of 211 DMGs were identified including all treatments and all contexts compared to control treatment. Twenty-five DMGs were identical in all three comparisons while 14, 20, and 33 DMGs in comparisons between control vs. D and control vs. US + D, control vs. D and control vs. US, and control vs. US and control vs. US + D, respectively (Figure 3a). A total of 122, 86, and 46 DMGs were affected in CpG, CHG, and CHH contexts, respectively (Figure 3b). Interestingly, no DMG was detected in the CHH context, which would have been identical in comparison of either the control vs. US or control vs. US + D with the control vs. D. However, in CpG and CHG contexts, 16 and 2 DMGs, respectively, were identified to be identical in all three comparisons (Figure 3b).

Using GO annotation biological processes (BP), cellular components (CC) and molecular functions (MF) were identified. When D was compared to the control, the DMGs were related to BPs of cellulose microfibril organization, cell growth and vesicle fusion with the Golgi apparatus in the CpG context, translation, BPs of interstrand cross-link repair, photosynthesis, photosynthetic electron transport in photosystem II and photosynthesis light reactions in the CHG context while only BP of photosynthesis in CHH context. For the cellular components, the DMGs were related to chloroplast and heme binding in the CpG context, to ribosome, chloroplast, Fanconi anaemia nuclear complex, extrinsic component of membrane, photosystem II oxygen evolving complex, photosystem II and I and thylakoid in the CHG context while photosystem I in CHH contexts. DMGs related to molecular functions were heme binding and oxidoreductase activity (acting on paired donors, with incorporation or reduction of molecular oxygen) in the CpG context, structural constituent of ribosome, electron transporter (transferring electrons within the cyclic electron transport pathway of photosynthesis activity) and NADH dehydrogenase (ubiquinone) activity in CHG context, while calmodulin binding in CHH context (Supplementary Tables 7−9).

Comparing US to the control, BPs of translation, photosynthesis, photosynthetic electron transport in photosystem II, cytochrome complex assembly, photosynthesis light reaction and proteolysis were annotated in the CpG context while photosystem II stabilization in the CHG context and obsolete anchored component of membrane and mediator complex in the CHH complex (Supplementary Tables 7−9).

Analyzing the combined treatments (US + D), most BPs were also affected in the CpG context, i.e., cellulose microfibril organization, cell growth, vesicle fusion with the Golgi apparatus, photosynthesis, proteolysis, cytochrome complex assembly, dephosphorylation, translation, the chitin catabolic process, and the cell wall macromolecule catabolic process. In CHG context the photosynthesis while in CHH context, the cell cycle modulation was annotated. As for cellular components in CpG context obsolete anchored component of membrane, obsolete integral component of thylakoid membrane, chloroplast, Golgi membrane, and ribosome, while in CHG context photosystem II oxygen evolving complex and photosystem II were annotated. The most molecular functions, such as heme binding, iron ion binding, oxidoreductase activity (acting on paired donors, with incorporation or reduction of molecular oxygen), serine-type endopeptidase activity, protein tyrosine/serine/threonine phosphatase activity, GTPase activity, structural constituent of ribosomes, and chitinase activity, were affected in the CpG context. In CHG and CHH context only 2−2 molecular functions were annotated, i.e., calcium ion binding and ATP hydrolysis activity, as well as cyclin-dependent protein serine/threonine kinase inhibitor activity and calmodulin binding, respectively (Supplementary Tables 7−9).

When US, D, and US + D treatments were pairwise compared, a total of 189 genes had altered methylation at cytosine sites, 21, 33, and 13 of which were altered in both comparisons between US + D vs. D and D vs. US, D vs. US and US + D vs. US, as well as US + D vs. US and US + D vs. D, respectively. Ten DMRs were identical in all three comparisons (Figure 3c; Supplementary Tables 4−6). Most DMRs, a total of 103, were identified in CpG context and about half of which were detected in CHG (53 DMRs) and CHH (57 DMRs) contexts, respectively (Figure 3d; Supplementary Tables 4−6). 52, 35, and 32 DMRs were detected in CpG, CHG and CHH contexts, respectively in D vs. US comparison, of which 15, 35 and 32 occurred only in this comparison, i.e., either in US + D vs. D or US + D vs. US comparisons, respectively (Figure 3d; Supplementary Tables 4−6).

GO annotation revealed the BPs, CCs and MFs related to the comparison of D vs. US (Supplementary Tables 7−9). Most BPs were affected in CpG context, i.e., cellulose microfibril organization, cell growth, photosynthesis, vesicle fusion with the Golgi apparatus, photosynthetic electron transport in photosystem II, the chitin catabolic process and the cell wall macromolecule catabolic process. In CHG context, photosynthesis, photosystem II stabilization, cytochrome complex assembly and cell wall modification while in CHH context gluconeogenesis, microtubule-based movement, ubiquitin-dependent protein catabolic process, xenobiotic transmembrane transport and cell wall modification were affected. CCs were annotated in two contexts. In CpG context, CCs are related to obsolete anchored components of the membrane, obsolete integral components of the thylakoid membrane, Golgi membrane, extrinsic components of the membrane, photosystem II oxygen-evolving complex and photosystem II while in CHG context to photosystem II reaction center and photosystem II. In CHH context, no annotation could be made. MFs of serine-type endopeptidase activity, electron transfer activity, GTPase activity, iron ion binding, electron transporter (transferring electrons within the cyclic electron transport pathway of photosynthesis activity), chitinase activity and GTP binding were annotated in CpG context while ATPase-coupled transmembrane transporter activity and pectinesterase activity in the CHG context. Most MFs were related to DMRs affected in the CHH context, as follows: phosphoenolpyruvate carboxykinase (ATP) activity, acireductone dioxygenase [iron(II)-requiring] activity, ubiquitin protein ligase binding, microtubule motor activity, O-methyltransferase activity, microtubule binding, xenobiotic transmembrane transporter activity, antiporter activity, pectinesterase activity, and ATP hydrolysis activity (Supplementary Tables 7−9).

As for comparisons to US + D, in CpG context, BPs related to vesicle fusion with the Golgi apparatus, dephosphorylation, and chitin catabolic process in US + D vs. US comparison while BPs of photosynthesis, photosynthetic electron transport in photosystem II, photosynthesis, light reactions, vesicle fusion with the Golgi apparatus, translation, cellulose microfibril organization, cell growth, proton motive force-driven ATP synthesis and protein peptidyl-prolyl isomerization in US + D vs. D comparison were affected. In the CHG context, BPs related to photosystem II stabilization and photosynthesis in US + D vs. US comparison while those related to vesicle fusion with the Golgi apparatus, intracellular protein transport, and photosynthesis in US + D vs. D comparison were annotated. In CHH context, no BPs were annotated to DMGs. DMGs were related to the Golgi membrane in US + D vs. US comparison and to a series of cellular components (chloroplast, thylakoid, photosystem I, obsolete integral component of the thylakoid membrane, Golgi membrane, ribosome, extrinsic component of membrane, photosystem II oxygen evolving complex, and photosystem II) in US + D vs. D comparison in CpG context. In CHG context, the photosystem II reaction center and photosystem II in US + D vs. US comparison while Golgi membrane and cytoplasm in US + D vs. D comparison were annotated. In CHH context, no CC annotation was found. With respect to the CpG context, molecular functions related to protein tyrosine/serine/threonine phosphatase activity and chitinase activity were annotated in the US + D vs. US comparison while electron transporter, transferring electrons (within the cyclic electron transport pathway of photosynthesis activity), structural constituent of ribosomes and peptidyl-prolyl cis-trans isomerase activity in US + D vs. D comparison. In CHG context, 2 MFs could be annotated in US + D vs. D comparison, i.e., methyltransferase activity and ATP hydrolysis activity. However, no MF was annotated for US + D vs. US comparison. Only one MF, methyltransferase activity, could be annotated in US + D vs. D comparison in the CHH context (Supplementary Tables 7−9).

 Between the RNA-seq and WGBS data, i.e., gene transcriptional and DNA methylation changes, no direct correlation was detected (Supplementary Table 10).

### Selection of target genes for transcriptional memory studies and validation of DEG analysis with RT-qPCR

Two differentially expressed genes (DEGs) were chosen on behalf of investigating the effect of drought: the probable aquaporin TIP3−2-like (TIP) that was up-regulated and the transcription factor MYB24-like (MYB) that was down-regulated. To assess the effect of ultrasound, following the same logic, the chosen DEGs were protein EXORDIUM-like 5 (EX) that was up-regulated and the ARGOS that was down-regulated. In addition, the chlorophyllase−2 (CHL) gene was selected because it was up-regulated in each treatment, but there was no significant difference in expression intensity between treatments. In further experiments on plant memory TIP MYB, EX and ARGOS target genes were selected and used. However, the CHL gene was used only for validation.

The list of housekeeping and target genes and their respective primer pairs are shown in Table 1.

**Table 1.** Primer pairs for housekeeping and target genes. ‘F’ and ‘R’ indicate forward and reverse primers, respectively.

Locus identifier (NCBI)	Gene description	Abbreviations	The sequence of forward and reverse primers (5’-3’)	Amplicon length (bp)
LOC101268171	Exocyst complex component SEC3A	SEC	F – CTCCTCAGTGGACAATGCGT R – TCTTCCACAGCAGCACTGAC	208
LOC544055	Elongation factor 1-alpha	ELO	F – AAGAGGCCATCAGACAAGCC R – TGAGGCAACATAACCACGCT	267
LOC100736499	Glyceraldehyde 3-phosphate dehydrogenase	GAPDH	F – ATTCCATGGGCACAGACTGG R – GGGAGCAAGGCAATTTGTGG	225
LOC138336996	Ubiquitin-ribosomal protein eS31 fusion protein-like	UBI	F – CCACTCTCCATCTCGTGCTC R – GTGAGCCCACACTTACCACA	247
LOC100316893	Protein auxin-regulated gene involved in organ size	ARGOS	F – GACATGGAATCATCAGAGGCA R – TGGCAATGGTGGAAGCATCA	240
LOC101252337	Protein EXORDIUM-like 5	EX	F – ACTCCCTCCGAGAACACTATCTTC R – CAACGAAACAGTACTCCACCACT	241
LOC101248880	Transcription factor MYB24-like	MYB	F – AGGCAGATGGAATTGTGTTGC R – CGTTGTCAGTTCTCCCTGGCA	206
LOC101265579	Chlorophyllase-2	CHL	F – CAAATCCGTACTGCCGGAGA R – ACGAGGGATGTATTCGAGAACG	205
LOC101251154	Probable aquaporin TIP3-2-like	TIP	F – TAGTGATAGCGTTGGCGCAT R – GCCATCAGTAGCAAGCCTCA	206

Based on the assumption, that the expression of the reference gene remains relatively constant under different experimental conditions, the most adequate housekeeping gene was UBI that would allow accurate comparison of gene expression levels (Supplementary Table 1).

Both mRNA-seq and RT-qPCR showed the same direction – either up- or down-regulation of differential expression and differential expression logarithmic fold change (LFC) values of the target genes as estimated by RT-qPCR. All of the DEGs were detected as true positive up- or down-regulated DEGs. The Pearson correlation coefficient was 0.88 (Supplementary Table 1). This high correlation coefficient indicates a strong positive correlation between the mRNA-seq LFC and RT-qPCR LFC.

### Formation and maintenance of somatic memory and the effect of stimulus repetition on memory maintenance

**Figure 4.**
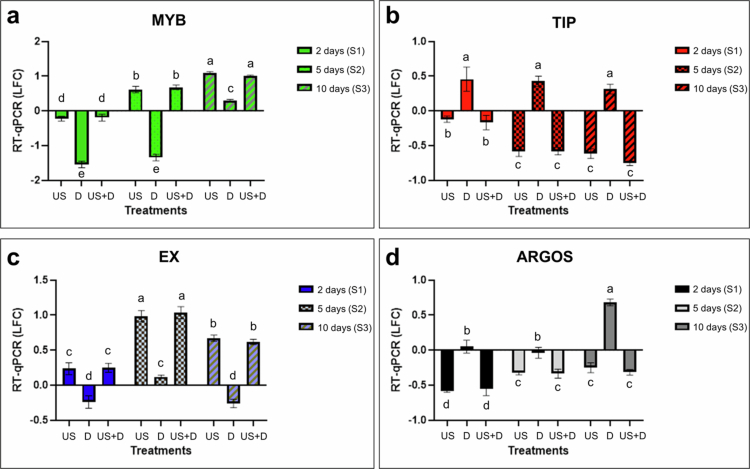
Memory maintenance for 5 or 10 d after stress stimulus. Differential expression logarithmic fold change values of target genes at 2 d (S1), 5 d (S2) and 10 d (S3) As estimated by RT-qPCR. Abbreviations: US – ultrasound treatment; D – drought treatment; US + D – combined ultrasound and drought treatment; LFC – logarithmic fold change compared to absolute control (c). All values were measured 2 d, 5 d or 10 d after a single treatment of 3-week-old plants. Error bars represent the standard deviation of the mean. Different lower-case letters above the error bars indicate significant differences at the 0.05 level (two-way ANOVA and post hoc Tukey’s B test results).

Based on the changes in the expression intensity of the selected target genes (TIP, MYB, EX and ARGOS), we examined the temporal duration of somatic memory retention and the effect of signal repetition given at different times on memory maintenance. To illustrate the maintenance of somatic memory, we plotted LFC values from RT-qPCR analysis of selected target genes at 2 d (S1), 5 d (S2) and 10 d (S3) (Figure 4; Supplementary Table 1) compared to the absolute control group at all sampling times (C at S1, ‘C and C’ at S2 and S3, respectively).

Under drought stress, the MYB gene was down-regulated to the same extent (no significant difference was observed) at sampling times of 2 d (D, S1) and 5 d (D, S2). In contrast, after 10 d, there was a significant difference between the expression intensity of MYB measured at the 10^th^ day (D, S3) compared to the LFCs of 2 d (D, S1) and 5 d (D, S2) samples (Figure 4a; Supplementary Table 1).

In the case of TIP, the gene expression was up-regulated in all three sampling times after the initial drought stress, and their values did not differ significantly from each other (with LFC values of 0.46, 0.43, and 0.31, respectively, at S1, S2, and S3) (Figure 4b; Supplementary Table 1).

Concerning EX, when US treatment was applied (either alone or in combination with D), up-regulation was observed at all three sampling times, with the highest positive LFC value found at S2, and a somewhat lower, but still positive LFC value at S3 compared to S1 (Figure 4c; Supplementary Table 1).

Whereas for ARGOS, when US treatment was applied (either alone or in combination with D), down-regulation was observed, again at all three sampling times, but with the lowest value found at S1, then the measured LFC values gradually increased over S2 and S3 (Figure 4d; Supplementary Table 1).

A significant correlation was detected using Pearson correlation between samples from 2 d (both mRNA-seq data and RT-qPCR data, respectively) and 5 d RT-qPCR data (*r* = 0.757, *r* = 0.748, respectively). The correlation was slightly lower (*r* = 0.647) but still significant when comparing 5 and 10 d RT-qPCR data (Supplementary Table 1).

**Figure 5.**
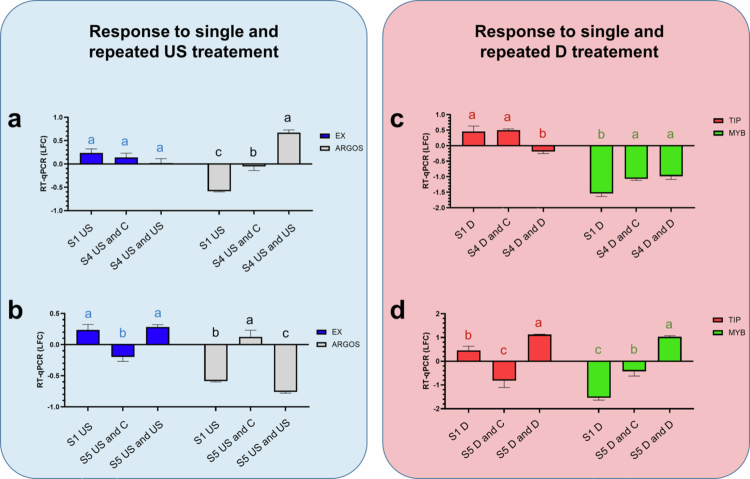
Effect of single and repeated exposure to stress stimuli on expression intensities of target genes at two time points. Differential expression of target genes (logarithmic fold change (LFC) values) at 2 d (S1), 7 d (S4) and 12 d (S5) by different treatments (ultrasound and drought stress), as assessed by RT-qPCR. All LFC values were in comparison with the absolute control group (C at S1; C and C at S4, S5). Abbreviations: S1 US – value measured 2 d after ultrasound treatment (S1); S4 US and C – value measured 7 d (S4) after single ultrasound treatment; S5 US and C – value measured 12 d (S5) after single ultrasound treatment; S4 US and US – value measured 7 d (S4) after initial application of ultrasound stress and 2 d after repeated ultrasound stress; S5 US and US – value measured 12 d (S5) after initial application of ultrasound stress and 2 d after repeated ultrasound stress; S1 D – value measured 2 d (S1) after applying single drought stress; S4 D and C – value measured 7 d (S4) after applying single drought stress; S5 D and C – value measured 12 d (S5) after applying single drought stress; S4 D and D – value measured 7 d (S4) after initial application of drought stress and 2 d after repeated drought stress; S5 D and D – value measured 12 d (S5) after initial application of drought stress and 2 d after repeated drought stress. Error bars represent the standard deviation of the mean. Different lower-case letters above the error bars indicate significant differences at the 0.05 level (two-way ANOVA and post hoc Tukey’s B test results).

 The EX gene showed an increase in expression after ultrasound treatment at 2 d (S1), which was maintained for 7 d (S4), as there was no significant difference between the two samples. However, when the same treatment (US) was repeated on day 5 (S2), the gene expression decreased by the 7^th^ day (S4) (Figure 5a). After 12 d, the gene expression intensity was further reduced and even down-regulated, but when the same treatment was applied on day 10 (S3), up-regulation was again observed (S5) and showed the same level as was detected at 2 d (S1) (Figure 5b, Supplementary Table 1).

The expression of ARGOS was significantly up-regulated 7 d after ultrasound treatment at S4 compared to the LFC value of the US treatment group at S1. However, the LFC remained negative. On the contrary, in response to a repeated US treatment on the 5^th^ day, it was up-regulated (S4) reaching a positive LFC (Figure 5a). Conversely, by the 12^th^ day, it was down-regulated by a repeated US treatment (S5) when the stimulus repetition was applied on the 10th day (S4). The expression level was significantly lower than the LFC achieved in the 2-day US group (S1) (Figure 5b, Supplementary Table 1).

The response to drought stress was maintained for 7 d (S4), with an up-regulation of the target gene TIP. A significant reduction was observed in expression intensity on the 7^th^ day (S4) following a repeated D treatment on the 5^th^ day (Figure 5c). However, after 12 d, TIP was significantly down-regulated without repeated drought (S5). On the contrary when the plants were re-treated on the 10^th^ day with drought stimulus, then, after 12 d (S5), gene expression intensity was significantly greater than that of the drought-treated plants at 2 d (S1) (Figure 5d).

When drought stress was applied, MYB was initially down-regulated (S1; D); however, the level of down-regulation decreased significantly by day 7 (S4; D and C). (Figure 5c). Repeated exposure to drought (D and D, S4) did not elicit any further changes in the expression levels of MYB, as there was no significant difference between these two treatment groups at S4. Similarly, as was detected on the 7^th^ day, after 12 d (S5; D and C), a decrease in the level of down-regulation was observed. However, after re-exposure to drought, an up-regulation of the target gene with a positive LFC value (S5; D and D) was detected (Figure 5d).

### Association-forming ability of tomato plant

If CS elicited the same gene expression response as was detected in response to USC after combined training (US + D treatment), it was considered association formation. In the case of TIP and MYB, US, while in the case of EX and ARGOS, D was considered as CS. To illustrate the results more precisely in a comprehensive manner, the relative gene expression intensities (LFCs) were calculated using the absolute control group for S1 samples and ‘US + D and C’ as controls for the S4 samples.

**Figure 6.**
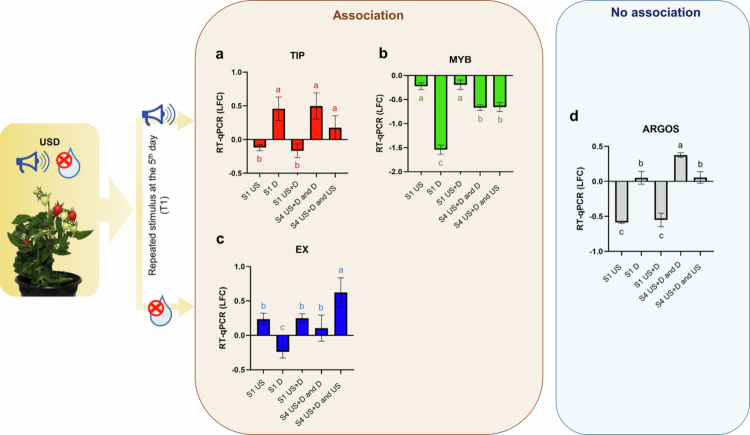
Changes in the expression intensities of target genes to stimulus repeat after combined training (conditioning). Differential expression logarithmic fold change values of target genes at 2 d (S1, compared to the absolute control, C), 5 d (S2, compared to their respective control group, US + D and C = value measured 7 d after initial application of combined ultrasound and drought stress) and 7 d (S4, compared to their respective control group, US + D and C = value measured 7 d after initial application of combined ultrasound and drought stress) as estimated by RT-qPCR. Abbreviations: S1 US – value measured 2 d after ultrasound treatment (S1); S1 D – value measured 2 d after drought treatment (S1); S1 US + D – value measured 2 d after a single, combined ultrasound and drought treatment (S1); S4 US + D and D – value measured 7 d (S4) after initial application of combined ultrasound and drought stress and 2 d after repeated drought stress; S4 US + D and US – value measured 7 d (S4) after initial application of combined ultrasound and drought stress and 2 d after repeated ultrasound stress; LFC – logarithmic fold change. Error bars represent the standard deviation of the mean. Different lower-case letters above the error bars indicate significant differences at the 0.05 level (two-way ANOVA and post hoc Tukey’s B test results).

The investigation of the expression levels of target genes at the 7^th^ day (S4) was conducted on tomato leaf samples using RT-qPCR in response to either US or D re-exposures, after combined training (US + D) at the start of the experiments. Re-exposure to US treatment on the 5^th^ day, after combined training (US + D), the results indicated a significant up-regulation of TIP (Figure 6a) and a significant down-regulation of MYB (Figure 6b) compared to non-retreated samples (US + D and C). The expression intensities of treatments when the re-treatment was made either by D or US (US + D and D, US + D and US), did not differ significantly from each other and showed the same tendency whether we look at TIP or MYB. The expression levels of EX were also investigated (Figure 6c). There was a significant increase in the LFC value after US + D-trained plants that were re-treated with US (US + D and US); however, re-treatment with D (US + D and D) also caused the up-regulation of EX compared to the LFC value of non-retreated plants (US + D and C). The expression levels after ‘US + D and D’ treatment (S4) differed significantly from the LFC value measured 2 d after a single D treatment (S1), but there was no significant difference between US (S1) and ‘US + D and D’ (S4). With regards to ARGOS (Figure 6d), ‘US + D and D’ treatment, when US + D-trained plants were re-treated with D on the 5^th^ day, showed a significant increase of the expression intensity compared to the values (S4) detected in non-retreated (US + D and C) and with US re-treated plants (US + D and US) (Figure 6; Supplementary Table 1).

## Discussion and conclusions

Our hypotheses included the effects of US and D applied single or in combination on changes on the global gene transcription and DNA methylation, as well as the correlation between them, the maintenance of transcriptional memory and the association-forming ability of plants.

### Global changes in gene transcription and DNA methylation and their correlation

When an environmental stimulus hits a plant, after recognizing and perceiving it, the plant responds to the stimulus. However, at the same time, it also begins to build memories about the stimulus which can later develop into either short-term somatic memory or long-term, even heritable memory. As a result, a primed plant becomes capable of coping with recurring stress events. The response, adaptation, and memory of plants to environmental abiotic and biotic stresses are accompanied by changes in their transcriptomic and epigenetic mechanisms.^1^^,^^4^^,^^10^^,^^11^^,^^12^^,^^55^

In this study, the effects of two abiotic stressors, the ultrasound and drought, were assessed on gene transcription and DNA methylation 2 d after their single or combined application to tomato plants. The revealed transcriptomic changes related to various biological processes, cellular components and molecular functions affected mainly the growth and stress processes, but the induced transcriptomic responses depended on the type of stressor (Figure 2, Supplementary Tables 2−3). These findings were consistent with previous transcriptomic studies of different plant species exposed to ultrasound^22^^,^^47^ and drought.^56^^,^^57^

 Similarly, both stimuli caused altered global DNA-methylation in the tomato plants (Figure 3). Most of the DNA methylation changes occurred in the CpG contexts, while the fewest occurred in CHH context (Supplementary Tables 4−6). Several growth- and stress-related processes, components, and functions, particularly those related to various membrane functions, photosynthesis, and translation, were affected in response to both stimuli. Although the changes occurred in similar processes, the changes that occurred were stress-type-dependent (Supplementary Tables 7−9). Similar processes were revealed in a previous study.^58^ As summarized in the study of Guarino et al.^59^, drought stress can cause changes in DNA methylation in various crops, such as *Medicago truncatula* Gaertn., *Oryza sativa* L., and *Triticum aestivum* L., both in CXG and CHH contexts. Regarding ultrasound Hidvégi et al.^60^ described DNA methylation changes in response to ultrasound in *T. aestivum* seedlings, whose methylation changes were correlated with the changes detected in gene transcription.

However, in our present study, we did not find a direct correlation, i.e., in either case, i.e., in response to drought or ultrasound, between the changes in gene transcription and DNA methylation. This is not a surprising result, considering that other molecular mechanisms are also involved in the formation of plant memory and different molecular layers of the plant memory cooperate with each other and thereby affect the transcriptomic response. ^11^^,^^12^^,^^15^ In response to drought, other epigenetic mechanisms, such as histone methylation (H3K4me2, H3K4me3, H3K27me2; H4R3sme2), acetylation (H3K9ac; H4ac; H4K5/8/12/16ac) and phosphorylation (H3T3ph) (reviewed in Guarino et al.^59^), as well as non-coding RNAs,^61^ including microRNAs,^62^ have also been implicated in stress memory. Thus, for further analysis, we relied on the results gathered from the RNA-seq analysis.

### Investigation of transcriptional memory formation and maintenance for four target genes

Plant transcriptional memory relies on modified transcriptional responses. Responsive genes are produced at a certain level following an initial stimulus, after which the recovery phase begins where the transcript levels may or may not return to baseline (pre-stress state) depending on the type of transcriptional memory. If the plant receives the same stimuli (both in strength and duration) and shows significantly different transcriptional levels of the same responsive genes, the criteria of transcriptional memory have been reached.^63-65^ The transcriptional-based somatic memory of selected genes was assumed if the direction of expression, i.e., up- or down-regulation (based on LFC) remained the same as was also detected in the case of S1 (2 d) and the LFC significantly differed from other treatments. As there were no significant differences between the measured LFC values of target genes between the US and US + D treatments in all target gene contexts, we established that the two stimuli (US and D) were truly independent of each other (Figure 4). Therefore, it is sufficient to examine the response to only the single and repeated stimulus that the target gene was chosen for in the first place (D for TIP and MYB and US for EX and ARGOS).

In the case of MYB, there was no significant difference between the transcriptional response to the initial stimuli (D, S1) and the gene expression intensity at day 5 (D, S2), which suggests that the plants retained memory for 5 d. The type of transcriptional memory depends on whether the transcript levels return to baseline.^63-65^ In this case, the duration of gene expression still exceeded the initial stimulus and the LFC values differed from the baseline resulting in hysteresis; therefore, Type 1 (or sustained induction) memory was observed. In contrast, after 10 d, the measured gene expression intensity values (D, S3) differed significantly from the previous measurements (D, S1 and S2); therefore, it seemed that transcriptional memory faded between 5 and 10 d (Figure 4a). More precisely, on day 7, the expression intensity of MYB was still differently expressed from the absolute control group (C and C, S4) but to a lesser extent; therefore, memory was still maintained (Figure 5c). This is also supported by the observation that when the plants were re-treated on day 5 (S2), there was no significant difference between the LFC values measured for ‘D and D’ and ‘D and C’ treatment groups at S4, suggesting that since the memory was maintained, repeated exposure to D did not elicit any additional particular reaction. However, when the re-exposure to the initial stimuli occurred on day 10 (S3), contrary to all our previous measurements of MYB expression intensity, we observed a positive LFC value for ‘D and D’ treatment groups. This supports our earlier suggestion that memory maintenance lasted only for 7 d, as we obtained significantly different responses to the repeated stimulus on day 12 (D and D, S5; Figure 5d). The up-regulation of MYB genes on the 12^th^ day may be due to the different physiological ages of the plants.

 Similarly, to MYB, TIP also showed the possibility of Type 1 transcriptional memory, but unlike the sustainability of MYB, the induction of TIP remained the same for not only 5 d but also 10 d (with no significant difference between the measured LFC values of D treatment group at S1, S2 or S3) (Figure 4b). However, at day 12, TIP was significantly down-regulated compared to the previously mentioned LFC values, indicating that the memory of drought was not sustained for 12 d (D and C, S5) (Figure 5d). This probably explains the phenomenon that when the D treatment was repeated on the 5^th^ day, TIP was still up-regulated on day 7, when ‘D and D’ treatment group was compared to ‘D and C’ at S4, as the difference was still positive but to a lesser extent (Supplementary Table 1). It has shown possible signs of habituation/familiarization. However, since the memory was only maintained for 10 d (Figure 4b), a significant up-regulation of TIP was observed on day 12 (D and D, S5) compared to the value measured at 2 d (D, S1). Up-regulation was detected for ‘D and D’ (S5) similarly as at 2 d (S1), showing the same tendency, but the LFC values differed significantly (Figure 5c). It indicates that on the 12^th^ day, the plants reacted more sensitively to drought than before (D, S1). In summary, it seems that plants sustained the induction of TIP as a form of transcriptional memory throughout 10 d, during which, if the unpleasant stimulus was repeated, possible signs of habituation were observed, but if the same stimulus was repeated after the memory was erased, the expression response occurred again. However, the expression intensity was significantly greater on day 12 (S5) than when it was applied to 3-week-old plants. The difference between the expression intensities may be due to the different physiological ages of the plants.

The results of the expression intensities of EX measured for the US treatment group at S1 and S2 imply that the level of up-regulation increased from 2 d (S1) to 5 d (S2). However, at day 10 (US, S3), the EX target gene was still up-regulated, but to a lesser extent compared to the values measured for US groups at S1 and S2. This shows that the gene expression changes in response to US occurred with a time delay, but a prolonged effect was still observable after 10 d, suggesting the sustained induction of this target gene (Type 1 transcriptional memory) (Figure 4c). On the 7^th^ day (S4), there was no excessive reaction to the repeated stimulus (US or US) because there was no significant up-regulation of the EX target gene; therefore, the plants may have learned and memorized to ignore the harmless stimulus, and quietened down their response intensity (familiarization) (Figure 5a). However, on the 12^th^ day, there was a change in the direction of the reaction to US as the expression intensity of EX was down-regulated, suggesting that the memory of this stimulus was erased by day 12 (Figure 5b). This assumption is supported by the fact that repeated US treatment on the 10^th^ day caused an up-regulation of EX gene on day 12 (US and US, S5) that was not significantly different from the LFC values measured at 2 d (US, S1). In this case, contrary to TIP, the age of the plant did not affect the intensity of the response.

Regarding ARGOS, the measured LFC values at S2 and S3 for US treatment were not significantly different from each other, but differed significantly compared to the value measured for the US treatment group at S1 (Figure 4d). This result suggests that there may be a case of sustained induction observed for ARGOS in response to US treatment for 10 d. If the repeated US stimulus fell within this time interval, then the response intensity changed both in direction and strength (US and US, S4; Figure 5a). However, on day 12, the measured LFC value in response to US treatment (US and C, S5) was significantly different from the prior measurements (US at S1, S2 and S3). This observation suggests that the memory was erased by day 12 (Figure 5b). The repeated US stimulus elicited the similar response, i.e., down-regulation of ARGOS, as was detected at S1. The expression behavior of ARGOS in response to repeated US exposure might suggest Type 2 transcriptional memory (modified re-induction; -/+).^63-65^

Sometimes, memory maintenance could hinder the recovery process or slow down the development of the plant; other times (in the case of the recurrence of the unpleasant stimuli) it is advantageous to store the once acquired information to maximize the individual’s fitness in times of need. Studying the temporal aspects of memory, Ding et al.^66^ reported that previous exposure to unfavorable stimuli (drought) provided the opportunity for plants to memorize this experience with increased transcript levels of ‘two trainable’ genes for a short period of time (5 d) and after 7 d it was erased. This observation supports our results that plants are able to remember past events for a short (7−10 d) period of time on a transcriptomic level. Naturally, the age at which plants are exposed to stimuli may determine the duration of memory maintenance. To minimize the cost of future unfavorable stress responses via diverting resources for defense and growth, investing in long-term maintenance of memory may be beneficial for young plants.^67^^,^^68^ Furthermore, in trees, long-term memory that lasts through several months is known because it is beneficial for them to remember the feeding damage in the preceding year to improve their anti-herbivore defense.^69^^,^^70^ On the contrary, annual plants choose to invest mainly in short-term memory formation before flowering thus minimizing their costs, in order to successfully reproduce.^71^

In conclusion, the plants memorized the effects of the stimuli and maintained the changed gene expression of four target genes. The US stimulus was memorized by the ARGOS and EX genes for 10 d; however, the degree of expression intensities depended on the genes and the sampling time (5^th^ or 10^th^ d). For the D stimulus, the decreased expression intensity of MYB was maintained only for 7 d, but the increased expression intensity of TIP was maintained for 10 d. An investigation of the gene expression intensities of target genes revealed that the repeated application of the two stimuli, i.e., training for either drought or ultrasound, might cause altered behavior, such as habituation, Type 1 (sustained induction) or Type 2 (modified re-induction) transcriptional memory, depending on the genes and physiological ages of the plants.

#### Assessing the association-forming ability

Cross-acclimation is a phenomenon where exposure to a non-lethal stress results in tolerance to another stress leading to improving the individual’s fitness. Cross-acclimation relies on shared signaling pathways that may be activated by different types of stresses; furthermore, the interactions of the stresses are synergistic.^72^ However, to rule out the possibility of cross-priming, the target genes were chosen to show different expression patterns for the two signals (US and D), preferably either up- or down-regulation to one stimulus, and no response to the other based on the LFC measured after mRNA-seq at 2 d (S1). Association was assumed if, after combined training (US + D treatment), the conditioned stimulus (CS) elicited the same gene expression response as was detected in response to the other, unconditioned stimulus (UCS). Among the four target genes included in this part of our present study, three out of the four examined genes showed the possible ability of associative learning in plants, namely TIP, MYB, and EX (Figure 6; Supplementary Table 1).

The expression of TIP was significantly up-regulated in response to D at S1. After combined training (US + D) and then exposing the plants to the initially neutral cue, the ultrasound, it elicited an up-regulation of TIP. The LFC did not differ significantly either from the LFC of D treatment measured at S1 and LFC of ‘US + D and D’ treatment (repetition of D after combined training) at S4. Therefore, it evoked a conditioned response (CR). Although there was a difference in values between the expression intensities, the two groups (US + D and D, US + D and US) did not differ significantly from each other. This observation suggests that the plants were able to memorize this association between the two stimuli due to combined training (US + D treatment), and achieved a suitable response at the transcriptomic level, i.e., to up-regulate the TIP after applying CS and ultrasound on the 5^th^ day (Figure 6a; Supplementary Table 1).

Following the same logic, ultrasound also served as a CS, as it had no effect on the gene expression of MYB at S1 according to the mRNA-seq data, but drought elicited down-regulation of the target gene. After conditioning (US + D), similarly to TIP, US also elicited the CR. The existence of associative memory may be assumed, as we measured the same expression intensity (no significant difference) when the conditioned plants received D treatment (US + D and D) or if they received US treatment (US + D and US) on the 5^th^ day (Figure 6b; Supplementary Table 1).

Based on the mRNA-seq data, D served as a neutral cue (no expression change compared to the control sample), and US functioned as an UCS because the US signal (alone or in a combination with D) caused a significant up-regulation of EX, and down-regulation of ARGOS.

The EX expression intensities resulting from ‘US + D and US’ and ‘US + D and D’ treatments, when either D or US was applied to US + D-trained plants, differed significantly from each other. Presumably, the difference is due to the delayed response to US, as EX was overexpressed on day 5 (S2) compared to the results measured at 2 d (S1). Regardless, at the 7^th^ day (S4), the D treatment of US + D-trained plants (US + D and D) caused the up-regulation of EX, suggesting that D elicited the CR and suggested an association formation (Figure 6c; Supplementary Table 1).

In contrast, regarding ARGOS, although we were able to demonstrate somatic transcriptional short-term memory maintenance in the case of the US and US + D treatment (Figure 4), we found no evidence of association formation between the two stimuli (US and D). Initially, the target gene was chosen based on the expression logarithmic fold change values of 2 d samples (S1) originated from mRNA-seq data, where D would have served as the neutral (CS) and US would have functioned as an UCS. Therefore, based on this hypothesis, we should have observed that by the 7^th^ day (S4), D would have elicited the CR (e.g., the down-regulation of ARGOS) after conditioning, but the results showed a different degrees and directions of expression levels (Figure 6d; Supplementary Table 1). Therefore, in that case no association formation can be suggested.

In summary, to examine plant associative transcriptional memory, it is not enough to combine the neutral (CS) and unconditioned signals UCS and then CS subsequently and examine the response of the plants. However, in parallel, we must also emphasize the examination of the degree of plant transcriptional memory, since it cannot be neglected that the memory maintenance and its degree influence the results of subsequent treatments. These observations may provide an explanation for the differences between TIP, MYB, and EX expression patterns. In addition, even if 3 out of the 4 target genes maintained their transcriptional memory for 5−10 d, associations could not be detected by the 12^th^ day (Supplementary Table 1).

Our findings on the association-forming ability of plants confirm previous studies on associative learning in plants by Bhandawat et al.^33^ and Gagliano et al.^28^ One of the major differences between the studies was linked to the duration of the training. Bhandawat et al.^33^ conditioned the plants for an hour (20 min of music, 20 min of music alongside heat stress then 20 min of heat), while Gagliano et al.^28^ trained the plants each day three times for 2 h (60 min of airflow, 30 min of airflow + light, 30 min of light) separated by 1 h intervals, and trainings were repeated for 3−5 d. However, in our present study, the treatments (both D and US) required only a fraction of the time of previous experiments and only a single training period was used; however, we were able to determine the association-forming ability of the plants. Nevertheless, further studies are needed to determine how the strength and type of a signal influence the training regimen and the time required for association formation, as well as the duration of association maintenance in plants.

In conclusion, after combined training the plants with two stimuli (US + D), the associative learning ability of tomato plants, i.e., one neutral stimulus (CS), elicited the same gene expression response as the other stimulus (UCS), became detectable. If one of the stimuli was applied 5 d after the training, plants seemed to be able to connect different stimuli and use one neutral stimulus as an indicator/predictor of the other one in the case of 3 out of the 4 target genes examined. US (as a CS) applied 5 d after combined training induced the same expression intensity changes as were induced by D, in the case of TIP and MYB. D (as a CS) stimulus applied 5 d after combined training (US + D) triggered the same expression intensity of the EX gene, as was induced by US. If the stimuli were applied 10 d after combined training, the association ability was not detectable. It seems that associative memory of the plant could only be maintained for a short time (for 7 d) in transcriptional memory; at least for the 4 target genes we studied.

In the future, the results we gained from the present study may be useful for more detailed studies examining the transcriptional behavior of a larger number of genes together with other epigenetic changes, such as histone modifications, or alterations in non-coding RNA population. Unraveling the still hidden abilities of plants based on the memory function, especially in the field of training and conditioning, can also contribute to sustainable agricultural utilization of plant memory.^15^^,^^73^^,^^74^

## Supplementary Material

Supplementary material **Supplementary Figure 1.** Principal component analysis of the RNA-seq results. Two-dimensional Principal Component Analysis (PCA) plot showing the grouping of control samples (C.1–C.3) in orange and treated samples: drought stressed (D.1–D.3) in green, ultrasound treated (US.1–US.3) in cyan and combined drought and ultrasound treated (US+D.1–US+D.3) in purple.

Supplementary material **Supplementary Table 1.** Comprehensive data set of RT-qPCR measurements.

Supplementary material **Supplementary Table 2.** Significantly differentially over- and under-expressed genes (DEGs) 2 d after single or combined application of ultrasound and drought stimuli to tomato plants, in pairwise comparisons.

Supplementary material **Supplementary Table 3.** Gene ontology (GO) enrichment of transcriptionally up- or down-regulated genes 2 d after single or combined application of ultrasound and drought stimuli to tomato plants, in pairwise comparisons.

Supplementary material **Supplementary Table 4.** Gene annotations of differentially methylated genes (DMGs) in the CpG context based on the GO annotation results 2 d after single or combined application of ultrasound and drought stimuli to tomato plants.

Supplementary material **Supplementary Table 5.** Gene annotations of differentially methylated genes (DMGs) in the CHG context based on the GO annotation results 2 d after single or combined application of ultrasound and drought stimuli to tomato plants.

Supplementary material **Supplementary Table 6**. Gene annotations of differentially methylated genes (DMGs) in the CHH context based on the GO annotation results 2 d after single or combined application of ultrasound and drought stimuli to tomato plants

Supplementary material **Supplementary Table 7.** Gene ontology (GO) enrichment of DMGs that were either hyper-, or hypomethylated in the CpG context 2 d after single or combined application of ultrasound and drought stimuli to tomato plants, in pairwise comparisons.

Supplementary material **Supplementary Table 8**. Gene ontology (GO) enrichment of DMGs that were either hyper-, or hypomethylated in the CHG context 2 d after single or combined application of ultrasound and drought stimuli to tomato plants, in pairwise comparisons.

Supplementary material **Supplementary Table 9**. Gene ontology (GO) enrichment of DMGs that were either hyper-, or hypomethylated in the CHH context 2 d after single or combined application of ultrasound and drought stimuli to tomato plants, in pairwise comparisons.

Supplementary material **Supplementary Table 10**. Correlation between gene expression and DNA methylation 2 d after single or combined application of ultrasound and drought stimuli to tomato plants.

## Data Availability

All raw sequencing data for both RNA-seq and WGBS have been deposited in the NCBI Gene Expression Omnibus (GEO) repository under the accession number GSE303939. Apart from that, all relevant data can be found within the manuscript and its supporting materials.
