# Supplementary figures and images for: Short-term transcriptional memory and association-forming ability of tomato plants in response to ultrasound and drought stress stimuli
Source: Plant Signal Behav. 2025 Sep 12;20(1):2556982. doi: 10.1080/15592324.2025.2556982 (PMC12456219; doi:10.1080/15592324.2025.2556982)

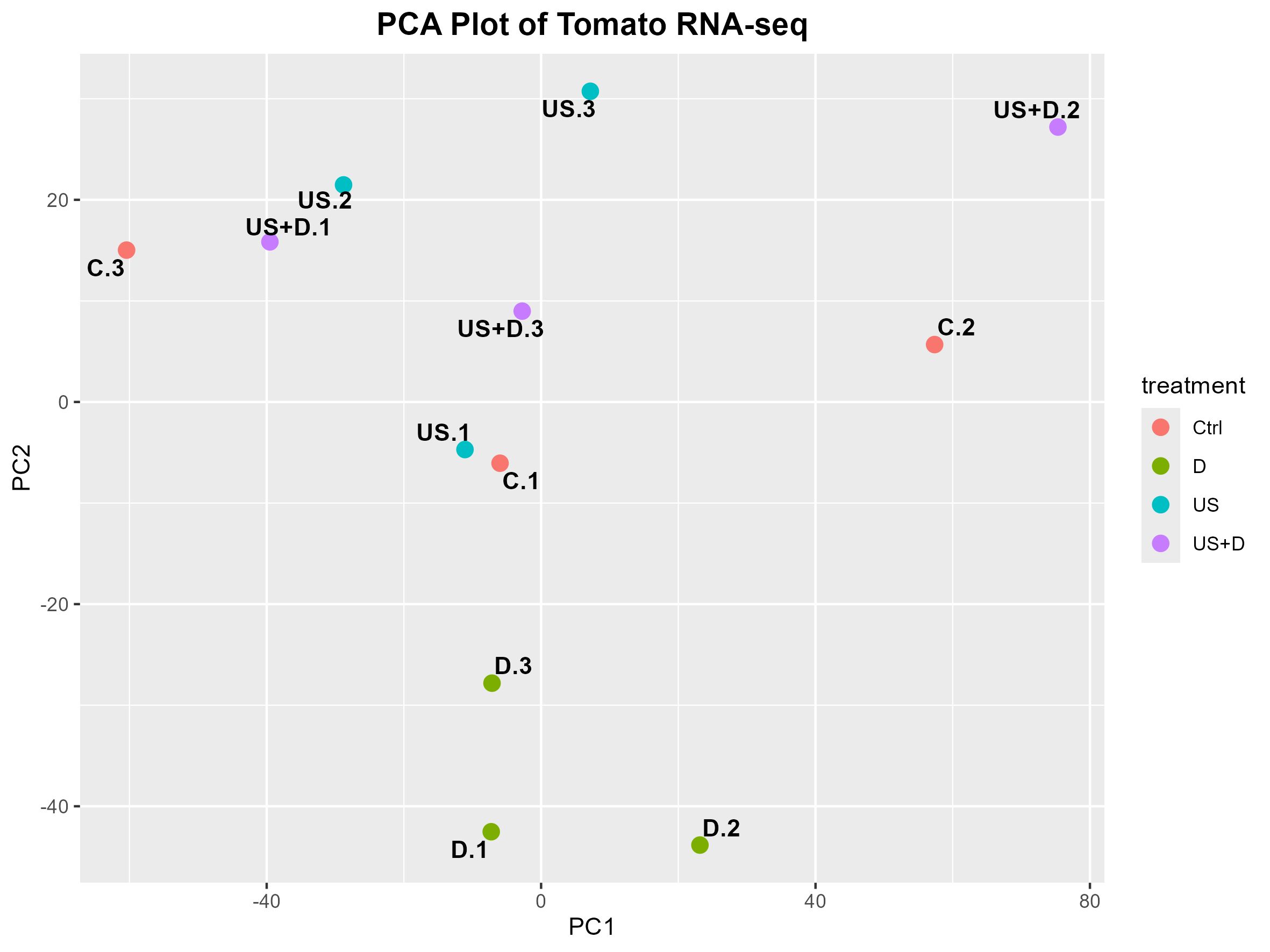

Supplement: Supplementary material — Supplementary Figure 1. Principal component analysis of the RNA-seq results. Two-dimensional Principal Component Analysis (PCA) plot showing the grouping of control samples (C.1–C.3) in orange and treated samples: drought stressed (D.1–D.3) in green, ultrasound treated (US.1–US.3) in cyan and combined drought and ultrasound treated (US+D.1–US+D.3) in purple. [file KPSB_A_2556982_SM0001.tif]
